# The complete mitochondrial genome of *Paranemachilus genilepis* (Zhu 1983) (Cypriniformes: Nemacheilidae) and its phylogenetic status

**DOI:** 10.1080/23802359.2020.1832931

**Published:** 2020-11-06

**Authors:** Congqiang Luo, Yusen Li, Pinhong Yang, Suqin Wang

**Affiliations:** a Hunan Engineering Research Center of Aquatic Organism Resources and Environmental Ecology, Hunan University of Arts and Science, Changde, China; b Hunan Provincial Key Laboratory for Health Aquaculture and Product Processing in Dongting Lake Area, Hunan University of Arts and Science, Changde, China; c Hunan Provincial Collaborative Innovation Center for Efficient and Health Production of Fisheries, Hunan University of Arts and Science, Changde, China; d Guangxi Key Laboratory of Aquatic Genetic Breeding and Healthy Aquaculture, Guangxi Academy of Fishery Sciences, Nanning, China

**Keywords:** Paranemachilus genilepis, mitochondrial genome, phylogenetic analyses

## Abstract

*Paranemachilus genilepis* (Zhu 1983) is a small and benthic loach species that mainly distributes in the Guangxi Province, China. To date, little was known about the genetic information of this species as no molecular sequence has been published. In this study, the complete mitochondrial genome of *P. genilepis* was reported using the Illumina MiSeq platform. The genome was 16,563 base pairs (bp) in length and its structure was identical to most genomes of bony fishes. Phylogenetic analyses supported two clades (I and II) among Nemacheilidae species and *P. genilepis* was sister to *Oreonectes furcocaudalis*.

## Introduction

*Paranemachilus genilepis* (Zhu 1983) (Nemacheilidae) is a small loach species that was mainly found in the Guangxi Province, China (Zhu 1989; www.fishbase.org). This species prefers to living in the underground stream and is called as cave species. To date, as no relative genetic analyses have been conducted for this species, we knew little about the genetic information of this species. Herein, we sequenced the whole mitochondrial genome of *P. genilepis* and then inferred its phylogenetic status.

The sample of *P. genilepis* was collected during July in 2019 at Fushui County (22.636 N, 107.898E), Guangxi Province, China. The sampled site locates in the Zuojiang River (a main tributary within the Pearl River). The specimen (Voucher number: FSYT2019001) was stored in the fish collection of Hunan University of Arts and Science. We obtained a bit of fin tissues and preserved them in 95% ethanol for DNA extraction. Total genomic DNA was extracted from fin tissues using a Genomic DNA Isolation Kit (QiaGene, Germany). The Illumina MiSeq platform (Illumina Inc, San Diego, CA, USA) was used to sequence the complete mitochondrial genome. We assembled the raw sequence reads into contigs using software SPAdes 3.9.0 (Bankevich et al. 2012). The final complete mitochondrial genome was produced using the contigs in SOAPdenovo (Luo et al. 2012).

The complete mitogenome sequence of *P. genilepis* (GenBank nos: MT845213) is 16,563 base pairs (bp) in length and is identical to other typical teleosts in the features of structural organization and gene order. That is, the mitogenome comprises of 13 protein-coding genes (*ND1*, *ND2*, *ND3*, *ND4*, *ND4L*, *ND5*, *ND6*, *COI*, *COII*, *COIII*, *ATP6*, *ATP8*, *Cyt b*), two rRNA genes (12S rRNA and 16S rRNA), 22 tRNA genes, and a control region (D-loop). We aligned the mitogenome of *P. genilepis* and 29 Nemacheilidae mitogenomes using MUSCLE (Edgar 2004). All 13 protein-coding genes were selected manually to combine into a concatenated sequence for phylogenetic analyses. Maximum likelihood (ML) technique with the optimal nucleotide substitution model (GTR + I + G) that was inferred from MRMODELTEST version 2.3 (Nylander 2004) were used for constructing trees. The ML tree was implemented in RAxML-VI-HPC (Stamatakis 2006) with 1000 nonparametric bootstrap replicates.

**Figure 1.**
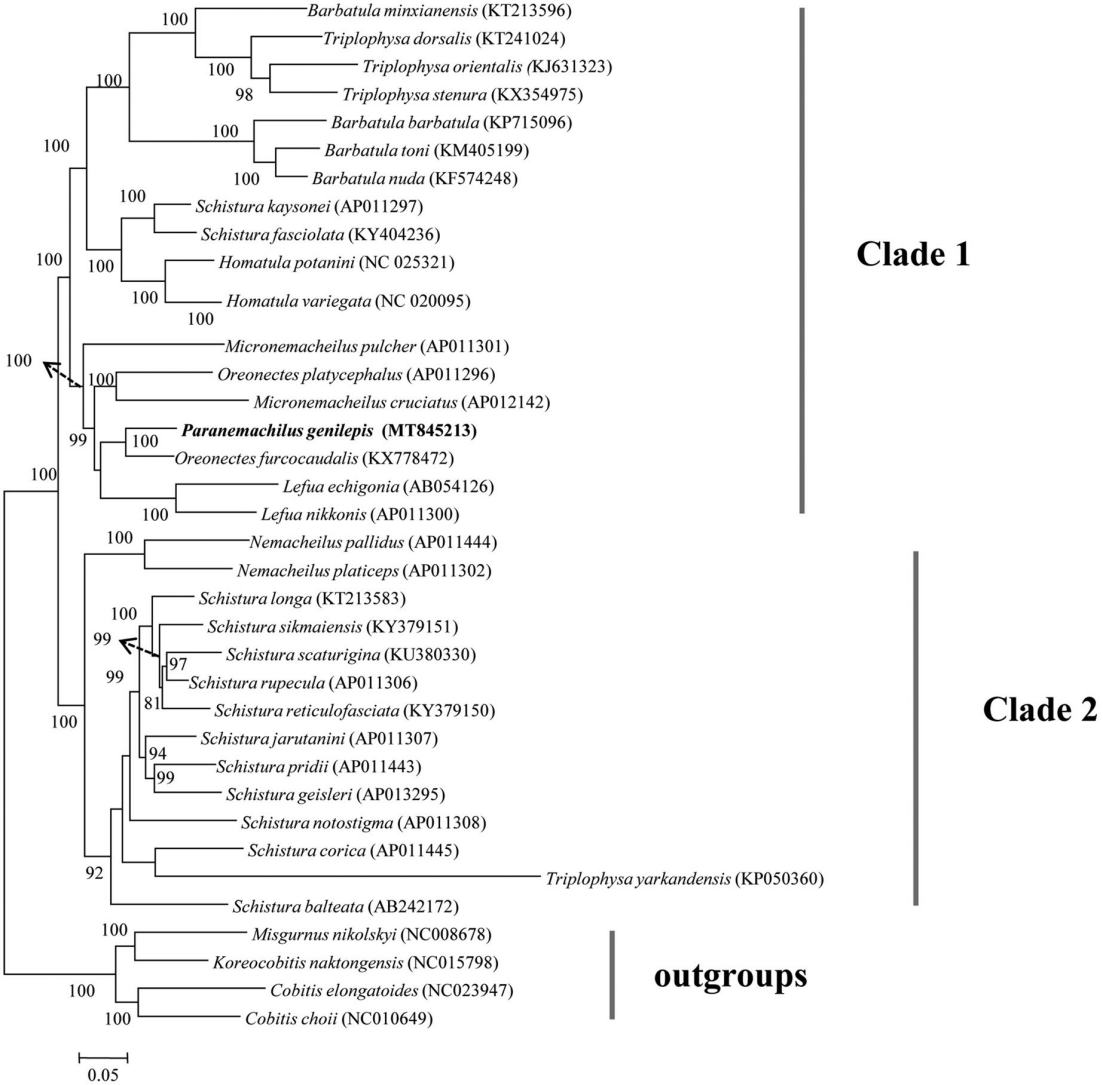
Maximum likelihood tree showing the relationships among 32 Nemacheilidae species based on 13 protein-coding genes. Values on branches indicate bootstrap values from maximum likelihood analyses.

ML tree yielded two well supported major clades with high supported values (Clade I and II; Figure 1). Clade I contained species from genera *Barbatula*, *Triplophysa*, *Schistura*, *Homatula*, *Micronemacheilus*, *Oreonectes*, *Lefua* and *Paranemachilus* (Figure 1). Clade II consisted of species from genera *Nemacheilus*, *Schistura* and *Triplophysa* (Figure 1). The cluster pattern implied taxonomic problems that occurred in genera *Schistura* and *Triplophysa*. *Paranemachilus genilepis* located in the clade I and was sister to the *Oreonectes furcocaudalis* (Figure 1). Taken together, more work on morphological traits and phylogenetic inferences is required to better understand the relationships among Nemacheilidae species.

## Ethical approval

Experiments were performed in accordance with the recommendations of the Ethics Committee of Hunan University of Arts and Science. These policies were enacted according to the Chinese Association for the Laboratory Animal Sciences and the Institutional Animal Care and Use Committee (IACUC) protocols.

## Data Availability

The data that support the findings of this study is openly available in GenBank of NCBI at http://www.ncbi.nlm.nih.gov, reference number MT845213.
